# Histone lysine-specific demethylase 1 induced renal fibrosis via decreasing sirtuin 3 expression and activating TGF-β1/Smad3 pathway in diabetic nephropathy

**DOI:** 10.1186/s13098-021-00771-z

**Published:** 2022-01-04

**Authors:** Lina Dong, Lei Yu, Jin Zhong

**Affiliations:** 1 Department of Nephrology, Inner Mongolia People’s Hospital, Hohhot, 010010 Inner Mongolia Autonomous Region China; 2 Department of Nephrology, Chongqing Hospital of Traditional Chinese Medicine, No. 6 Panxiqizhi Road, Jiangbei District, Chongqing, China

**Keywords:** LSD1, Renal fibrosis, Diabetic nephropathy, TGF-β1/Smad3 pathway, SIRT3

## Abstract

**Objective:**

Diabetic nephropathy (DN) is the leading cause of end-stage renal disease. Histone lysine-specific demethylase 1 (LSD1) is a flavin-containing amino oxidase that can repress or activate transcription. The aim of this study is to explore the mechanism of LSD1 aggravating DN-induced renal fibrosis.

**Methods:**

The STZ-induced DN rat model was established for in vivo study. The rats were divided into four groups: Sham, STZ, STZ + Ad-shNC and Ad-shLSD1. The Hematoxylin–eosin (HE) staining was used to evaluate the renal injury. The Immunofluorescence assay was used to determine the LSD1, Fibronectin and α-SMA expression. The related protein expression was detected by western blot.

**Results:**

Knockdown of LSD1 alleviated STZ-induced renal injury. Moreover, knockdown of LSD1 decreased the expression of serum biochemical markers, containing urine output (24 h), urinary protein (24 h), serum creatinine, BUN and UACR. Furthermore, we proved that knockdown of LSD1 alleviated renal fibrosis in STZ-induced DN rats. In vitro, knockdown of LSD1 suppressed NRK-49F cells activation and overexpression of LSD1 induced renal fibrosis. In addition, knockdown of LSD1 could deactivate TGF-β1/Smad3 pathway and promote sirtuin 3 (SIRT3) expression in vivo and in vitro. The rescue experiments confirmed that LSD1 induced renal fibrosis via decreasing SIRT3 expression and activating TGF-β1/Smad3 pathway.

**Conclusion:**

LSD1 deficiency leads to alleviate STZ-induced renal injury and overexpression of LSD1 induces renal fibrosis via decreasing SIRT3 expression and activating TGF-β1/Smad3 pathway, which provides a reasonable strategy for developing novel drugs targeting LDS1 to block renal fibrosis.

**Supplementary Information:**

The online version contains supplementary material available at 10.1186/s13098-021-00771-z.

## Introduction

Diabetic nephropathy (DN) is one of the main microvascular complications of diabetes, and it can cause end-stage renal disease, which is characterized by renal hyperplasia, basement membrane thickening and interstitial fibrosis [1]. The diabetes-induced metabolism and hemodynamics causes renal inflammation promotes the process from repair response to renal injury, and ultimately leads to renal fibrosis [2]. Although therapies have been established to control blood sugar and blood pressure, the number of patients who suffer from diabetic end-stage renal disease has increased year by year [3, 4]. Therefore, it is urgent to identify new pathologic mediators and therapeutic targets to prevent the progression of DN.

Epigenetic modifications mainly include genomic DNA methylation and histone modification. Histone methylation is a method of altering transcription by providing docking sites for chromatin modification instead of the charge of lysine [5]. Histone lysine residues can be monomethylated, dimethylated or trimethylated which are regulated by histone lysine methyltransferase and lysine demethylase [6]. Histone lysine-specific demethylase 1 (LSD1), also known as KDM1A, is a flavin-containing amino oxidase that specifically removes methyl groups from mono- and demethylated Lys4 and Lys9 of histone3 (H3K4Me1/2 and H3K9Me1/2) [7, 8]. H3k4Me1/2 is generally associated with transcriptional active genes, while H3K9Me1/2 is associated with transcriptional silencing [9, 10]. LSD1 is involved in various of biological processes, including cell proliferation, lipogenesis, and embryonic development [11–13]. In addition, LSD1 is considered to be an important epigenetic regulator of inflammatory responses in sepsis [14]. In hepatitis B virus-associated glomerulonephritis, LSD1 promotes renal inflammation by mediating TLR4 signaling pathway, and LSD1 positive is positively correlated with renal interstitial fibrosis [15]. Knockdown LSD1 can counteract the inhibitory effect of NR4A1 on TGF-β1-induced fibroblast collagen synthesis [16]. Thus, LSD1 plays an important role in organ fibrosis. More importantly, a recent study has reported that LSD1 activation contributes to pulmonary myofibroblast differentiation and fibrosis by targeting TGF-β1/Smad3 signaling [17]. However, the mechanism of LSD1 in DN-induced renal fibrosis has not been reported.

Transforming growth factor-β1 (TGF-β1)-Smad3 signaling pathway plays a central role in fibrotic kidney disease [18]. Overexpression of latent TGF-β1 in keratinocytes protects against renal fibrosis in an obstructive kidney disease model, indicating that overexpression of TGF-β1 exerts protective effect on renal injury [19]. SIRT3 is a major mitochondrial deacetylase and SIRT3 deficiency leads to the abnormal glycolysis of diabetic nephropathy [20]. SIRT3 inhibits fibrosis of renal tubule by preventing oxidative stress and mitochondrial dysfunction [21]. Activation of SIRT3 can improve cardiac fibrosis and cardiac function through the TGF-β1/Smad3 pathway [22]. Given the roles of TGF-β1/Smad3 pathway and SIRT3 in renal fibrosis, we hypothesized that LSD1 might play a vital role in DN-induced renal fibrosis through regulating SIRT3. This study aims to explore the role of LSD1 in diabetic renal fibrosis and provide a reasonable target for the diagnosis and treatment of DN.

## Methods and materials

### Animal experiments

The Sprague–Dawley (SD) male rats were purchased from Shanghai Animal Center (China) and the animal experiments were performed according to the Guide for the Care and Use of Laboratory Animals and approved by Inner Mongolia People’s Hospital [23]. At first, the rats were kept in a 12-h light/dark cycle and were given free access to food and water for 1-week adaptive feeding. After 12-h fasting, the STZ rats were received 55 mg/kg streptozotocin (STZ; Sigma-Aldrich, USA) intraperitoneal injection to establish STZ-induced DN rat models. After 7 days, the rat with fasting blood glucose levels from tail vein ≥ 16.7 mmol/L was considered a successful DN rat model. The Sham rats were received equal amount of sodium citrate (SC) as a control.

### Animal grouping

Three week later, a total of 10 Sham rats and 30 DN rats was assessed into four groups (10 per group):

Sham: The Sham rats were received 1 mL phosphate buffer saline (PBS) by tail vein injection for 1 week.

STZ: The STZ rats were received 1 mL PBS by tail vein injection for 1 week.

STZ + Ad-shNC: The STZ rats were received Ad-shNC (Genepharma, China) virus (1 × 10^11^ pfU virus dissolved in 1 mL PBS) by tail vein injection for 1 week.

STZ + Ad-shLSD1: The STZ rats were received Ad-shLSD1 (5′-GCCACCCAGAGAUAUUACU-3′, Genepharma) virus (1 × 10^11^ pfU virus dissolved in 1 mL PBS) by tail vein injection for 1 week.

After the 1-week injections, rats were maintained for 4 weeks. The urine output in 24 h was collected and measured. The rats were sacrificed under anesthesia at the end of the experiment. The blood samples were collected, and the serum was separated and stored at − 80 °C for further examination. Meanwhile, right renal tissues of rats were rapidly removed and stored at − 80 °C for further examination**.**

### Hematoxylin–eosin (HE) staining

The histopathological changes of rat renal tissues were observed by HE staining. After embedded with paraffin, the renal tissues were cut into sections. Then the sections were heated, dehydrated, cleared by xylene, and stained with hematoxylin (Solarbio, China). After dissimilated with hydrochloric acid alcohol, the ammonia water was added into the section to revert to blue again. Thereafter, the sections were dyed with eosin solution (Solarbio, China), dehydrated with gradient alcohol, and cleaned with xylene for twice. All sections were randomly evaluated under microscopy, and then the glomerular injury score and tubular injury score was calculated [24].

### Immunofluorescence assay

The paraffin-embedded sections of rat renal tissues were dewaxed and hydrated, and then the antigen was extracted with sodium citrate buffer at 98 °C for 20 min. Thereafter, the renal tissues or cells were fixed with 4% paraformaldehyde for 15 min at room temperature and then permeabilized with 0.5% Triton X-100 for 5 min at 25 °C. Afterwards, the sections were incubated with KDM1/LSD1 antibody (1/200; Abcam, UK), α-SMA antibody (1/200; Abcam, UK) or Fibronectin antibody (1/200; Abcam, UK) overnight at 4 °C. Then the sections were incubated with the Goat Anti-Rabbit IgG H&L (1/2000; Abcam, UK) or Goat Anti-Mouse IgG H&L (1/2000; Abcam, UK) at room temperature for 60 min. The nuclei were stained with DAPI and observed under a fluorescence microscopy.

### Enzyme-linked immunosorbent assay (ELISA)

The urinary protein (24 h), serum creatinine and blood urea nitrogen (BUN) were measured by Rat Urinary Protein ELISA kit (Shanghai Kang Lang Biological Technology co., LTD, China) and Rat serum creatinine ELISA kit (Renjiebio, China) and Rat Blood Urea Nitrogen ELISA kit (Jingmei biotechnology, China), respectively.

Finally, the urinary albumin-to-creatinine ratio (UACR) was calculated.

### Masson staining

The rat renal tissues were stained with Masson’s Trichrome Stain Kit (Solarbio, China). The renal tissues were deparaffinized, hydration, cut into sections and stained with hematoxylin for 8 min. Subsequently, the sections were incubated with ponceau acid fuchsin solution for 5 min and then washed with running tap water for 8 min. After differentiated with phosphotungstic acid for 5 min, the sections were transferred into aniline blue solution for 5 min. Finally, the sections were differentiated in 0.2% acetic acid for 2 min, following by dehydration, clearing, and mounting. The pathological morphology of the renal and expression of collagen fibers was observed under microscope. The results displayed that the collagen fibers were blue and muscle fibers were red.

### Cell culture and transfection

The rat renal fibroblast cell line NRK-49F cells were purchased from ATCC (USA) and cultured in DMEM supplemented with 10% FBS at 37 °C in 5% CO_2_. For transfection, the NRK-49F cells were transfected with Ad-shNC, Ad-shLSD1, SIRT3 (pcDNA3.0 recombined plasmid, Genepharma, China) or Ad-LSD1 by Lipofectamine 2000 (Thermo Fisher Scientific, USA). For co-transfection, the NRK-49F cells were co-transfected with Ad-LSD1 and SIRT3 with Lipofectamine 2000.

To study the effect of TGF-β1 on LSD1 expression in NRK-49F cells, cells were treated with 10 ng/mL recombinant human TGF-β1 (PeproTech, USA) for 72 h for further experiments.

### Western blot analysis

Total protein from tissues or cells was extracted by RIPA lysis buffer (Beyotime, China). Equal amount of protein was subjected in 10% SDS-PAGE (Beyotime, China) and then transferred onto PVDF membranes (Millipore, USA). After blocked in 5% skim milk, the membranes were incubated with one of specific primary antibodies overnight at 4 °C: KDM1/LSD1 antibody (1/600; Abcam, UK), α-SMA antibody (1/600; Abcam, UK), Fibronectin antibody (1/600; Abcam, UK), TGF-β1 (1/600; Proteintech, USA), Collagen I antibody (1/600; Abcam, UK), Collagen IV antibody (1/600; Abcam, UK), SIRT3 antibody (1/600; Abcam, UK), Smad3 antibody (1/600; Abcam, UK) and GAPDH (1/8000; Abcam, UK). After incubated with proper second antibody (Beyotime, China) for 1 h at room temperature, the ECL system (Beyotime, China) was used to detect the protein expression and GAPDH was used as control.

### Statistical analysis

All the results were presented as means ± SD and analyzed by Graphpad 7.0 (USA). Student’s *t*-test (two groups) and one-way ANOVA (no less than three groups) were used to analyze the differences among groups. A probability value less than 0.05 was considered statistically significant.

## Results

### Knockdown of LSD1 alleviates STZ-induced renal injury

**Fig. 1:**
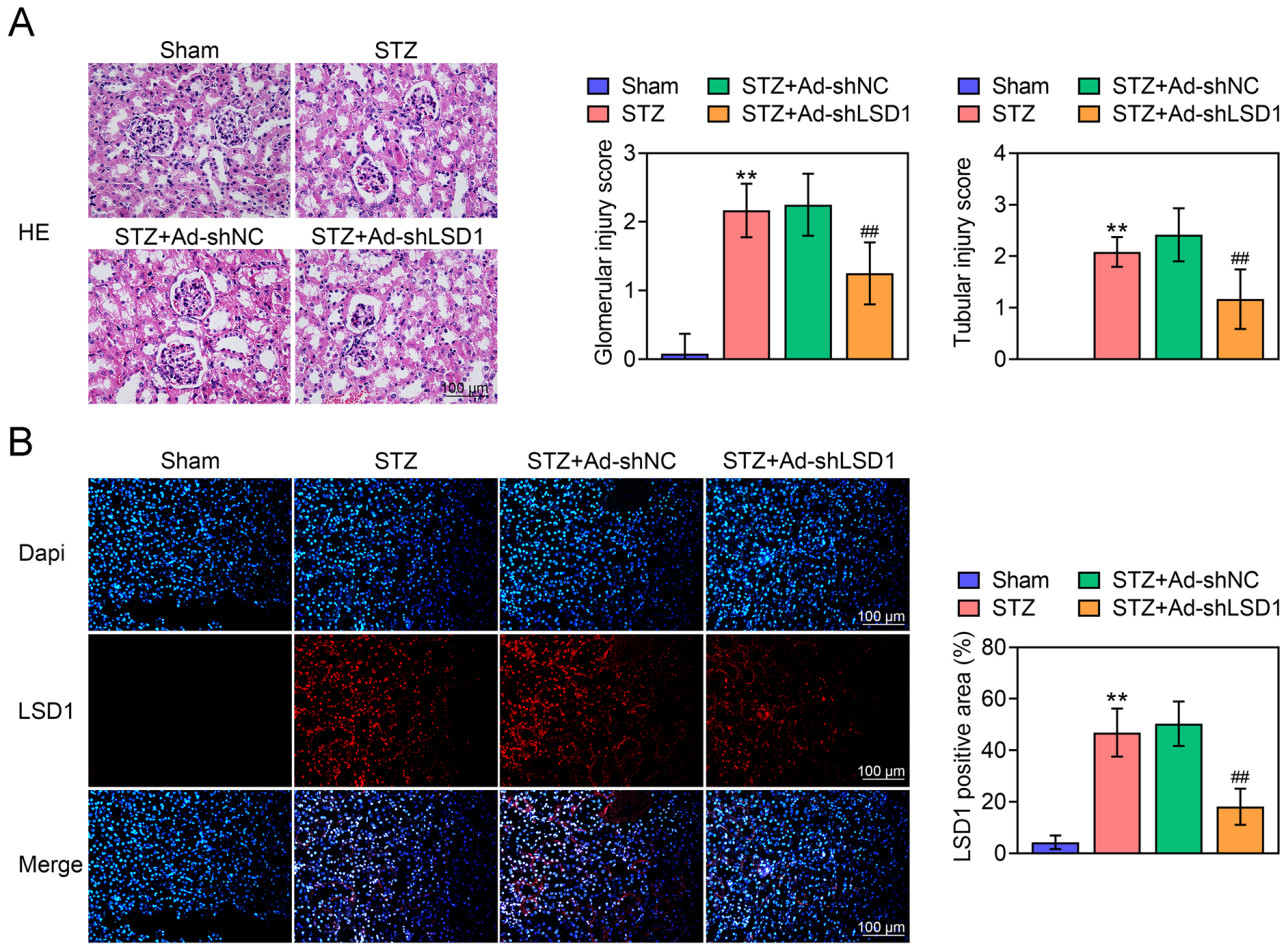
Knockdown of LSD1 alleviates STZ-induced renal injury. The STZ-induced DN rats were treated with Ad-shNC or Ad-shLSD1. **A** HE staining of the renal tissues of rats in each group indicated LSD1 alleviates STZ-induced renal injury (bar = 100 μm). **B** The LSD1 expression was detected by immunofluorescence assay (bar = 100 μm). Each group contained 10 rats. **p < 0.01 vs. Sham. ^##^*p* < 0.01 vs. STZ + Ad-shLSD1

To clarify the potential role of LSD1 in STZ-induced DN rats, the Ad-shLSD1 virus were injected in to DN rats. As shown in Fig. 1A the rats in Sham group presented normal size and morphology of glomeruli and renal tubules in renal tissues, accompanying with lower glomerular injury score and tubular injury score. Conversely, STZ-induced DN rats showed thinned renal cortex, expansive renal tubules and renal capsules, infiltrated tissue cells and hyperplastic interstitial fibroblasts in renal tissues, accompanying with higher glomerular injury score and tubular injury score. More importantly, knockdown of LSD1 significantly alleviated pathological changes and glomerular and tubular injury scores. Moreover, the IF staining indicated that Ad-shLSD1 markedly reduced the LSD1 expression in renal tissues of STZ-induced DN rats (Fig. 1B). These findings indicated that knockdown of LSD1 alleviated renal injury in STZ-induced DN rats.

### Knockdown of LSD1 decreases the expression of serum biochemical markers

**Fig. 2:**
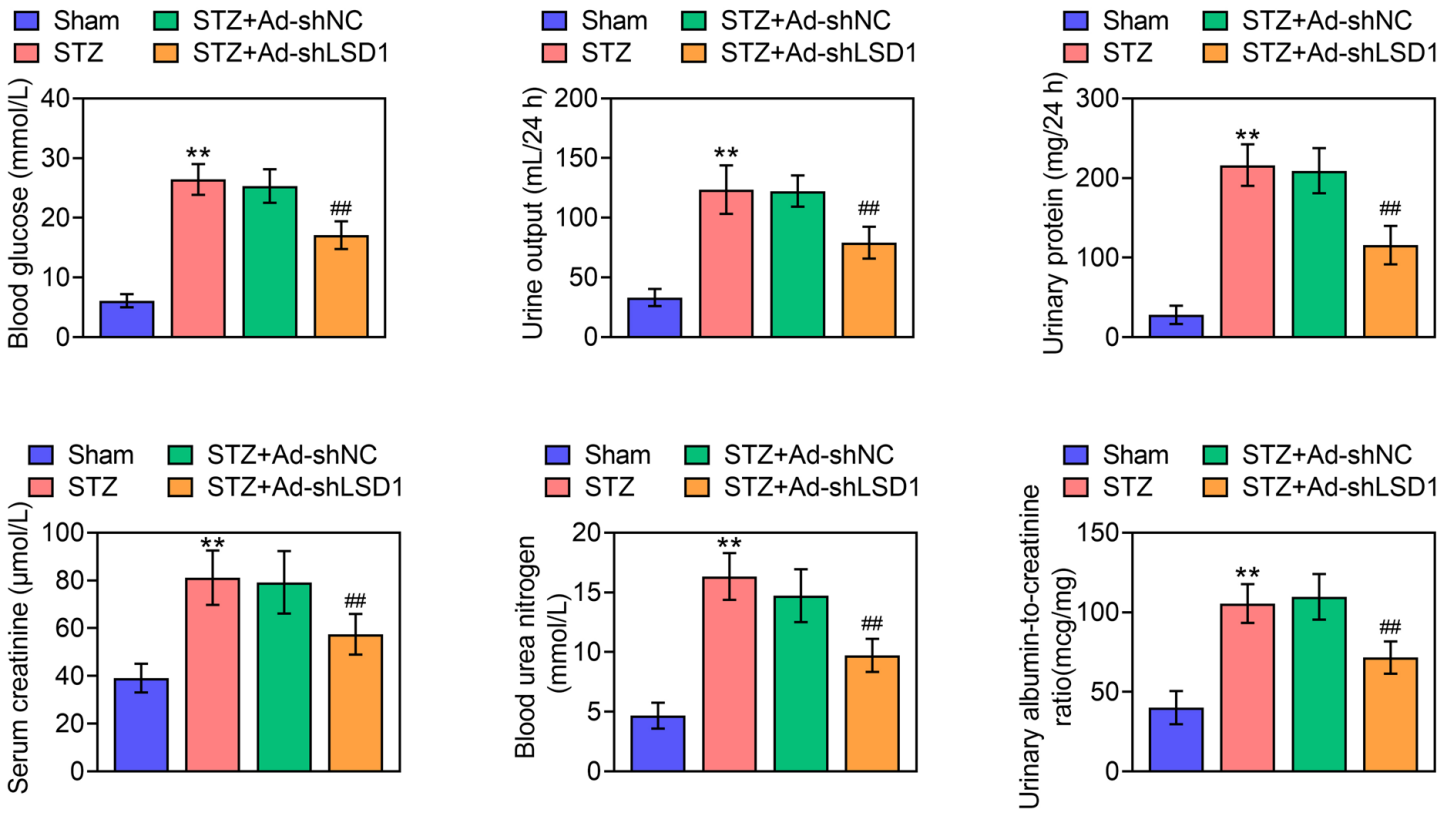
Knockdown of LSD1 decreases the expression of serum biochemical markers. The fasting blood glucose (FBG) was measured through blood from tail tip. The urine output in 24 h was measured. The urinary protein (24 h), serum creatinine and blood urea nitrogen (BUN) were determined by ELISA kit, respectively. The urinary albumin-to-creatinine ratio (UACR) was calculated. Each group contained 10 rats. **p < 0.01 vs. Sham. ^##^*p* < 0.01 vs. STZ + Ad-shLSD1

After injected with STZ, the blood glucose was ≥ 16.7 mmol/L, the urine output volume was greater than 50% of the rats Sham rats, the urinary protein was > 30 mg/24 h, indicating that the STZ-induced DN rat model was successfully established. More importantly, STZ increased the levels of serum creatinine, blood urea nitrogen (BUN) and urinary albumin-to-creatinine ratio (UACR) in DN rats. Fortunately, knockdown of LSD1 significantly reduced STZ-induced blood glucose, urine output (24 h), urinary protein (24 h), serum creatinine, BUN and UACR (Fig. 2). These results concluded that knockdown of LSD1 decreased the expression of serum biochemical markers in STZ-induced DN rats.

### Knockdown of LSD1 alleviates renal fibrosis in STZ-induced DN rats

**Fig. 3:**
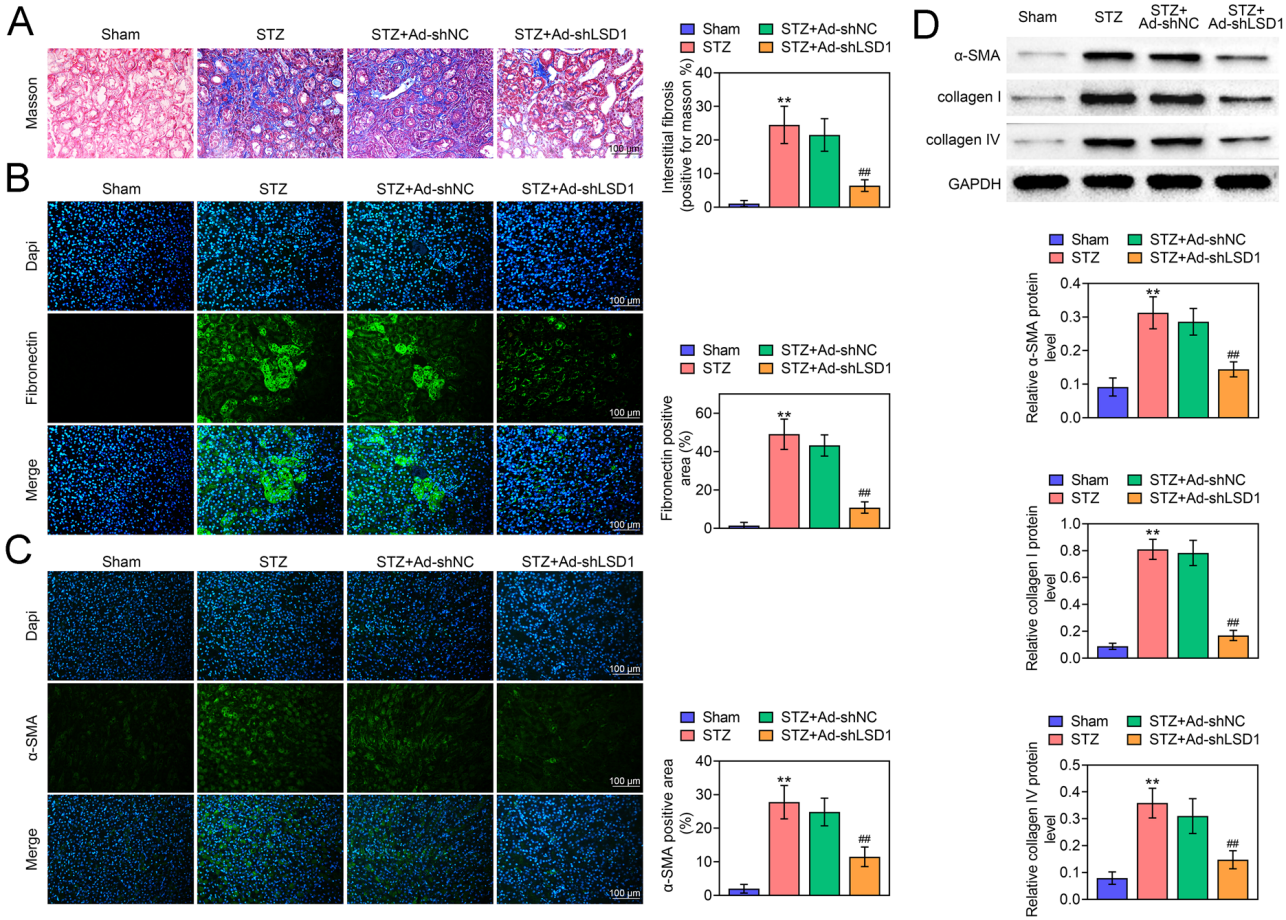
Knockdown of LSD1 alleviates renal fibrosis in STZ-induced DN rats. **A** Masson staining for renal fibrosis in different treated rats, suggesting that shLSD1 rescued STZ-induced renal fibrosis rats. The expression of Fibronectin (**B**) and α-SMA (**C**) were determined by IF assay, indicating shLSD1 reversed STZ-induced Fibronectin and α-SMA expression (bar = 100 μm). **D** The expression of α-SMA, collagen I and collagen IV were determined by western blot. Each group contained 10 rats. **p < 0.01 vs. Sham. ^##^*p* < 0.01 vs. STZ + Ad-shLSD1

In order to evaluate the impact of LSD1 on renal fibrosis, the Masson staining was performed. As shown in Fig. 3A, STZ-induced DN rats showed significant renal fibrils (blue) accumulation comparted with Sham rats. In addition, knockdown of LSD1 markedly reduced STZ-induced renal fibrils accumulation (Fig. 3A). Interstitial fibrosis is characterized by the production of interstitial matrix components such as collagen and the activation of α-SMA, accompanying with the activation of pro-fibrotic gene such as Fibronectin. Subsequently, the expression of Fibronectin (Fig. 3B) and α-SMA (Fig. 3C) were determined by IF staining, and the results indicated that STZ induced the expression and activation of α-SMA and Fibronectin, whereas these changes were reversed by knockdown of LSD1. In addition, the IF staining indicated that DN suppressed SIRT3 expression, and knockdown of LSD1 reversed this phenomenon (Additional file 1: Fig. S1D). Moreover, the western blot assay indicated that STZ-induced elevated expression of α-SMA, collagen I and collagen IV, whereas they were decreased by knockdown of LSD1 (Fig. 3D). More importantly, STZ treatment promoted TGF-ß expression, which was further decreased by sh-LSD1 in mice (Additional file 1: Fig. S1C). Thus, these findings concluded that knockdown of LSD1 alleviated renal fibrosis in STZ-induced DN rats.

### Knockdown of LSD1 suppresses NRK-49F cells activation

**Fig. 4:**
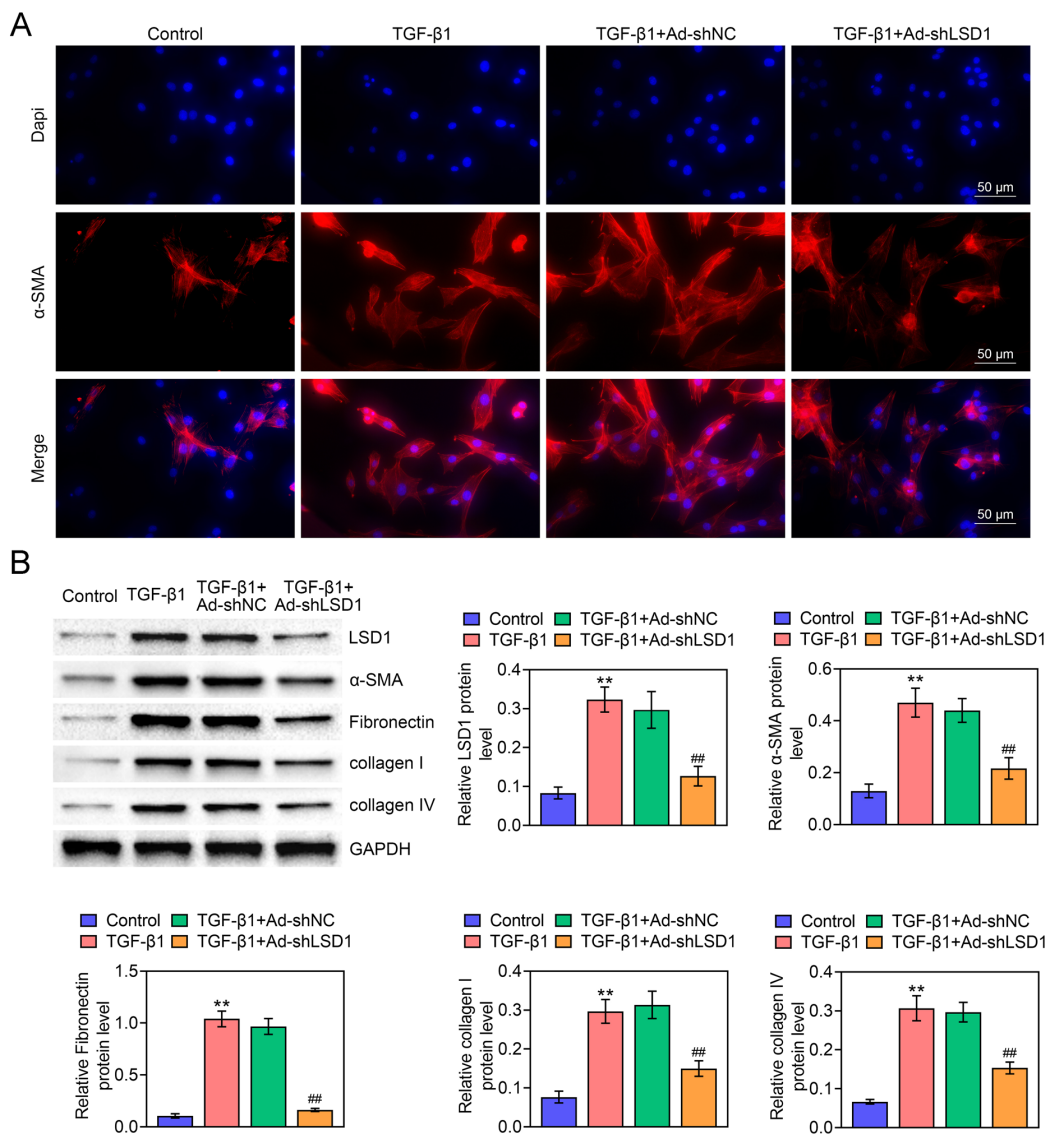
Knockdown of LSD1 suppresses NRK-49F cells activation. The NRK-49F cells were transfected with Ad-shNC or Ad-shLSD1 and then treated with 10 ng/ml TGF-β1 for 72 h. **A** The expression and location of α-SMA were detected by IF assay (bar = 100 μm). **B** The protein levels of LSD1, α-SMA, Fibronectin, collagen I and collagen IV were evaluated by western blot. Each experiment repeated three times. **p < 0.01 vs. Control. ^##^*p* < 0.01 vs. TGF-β1 + Ad-shLSD1

High glucose could induce renal fibroblast (NRK-49F) cell proliferation and activation to myofibroblasts in DN. Therefore, the activation of NRK-49F cells plays a vital role in DN [25]. In cells, overproduction of TGF-β1 contributes to rapid renal fibrosis which can mimic renal fibrosis diseases [26]. To verify whether LSD1 participates in TGF-β1-stimulated NRK-49F fibroblast activation, the NRK-49F cell transfected with Ad-shNC or Ad-shLSD1 were treated with TGF-β1, and then the α-SMA expression was determined by IF assay. As Fig. 4A displayed, TGF-β1 induced the expression and activation of α-SMA. Diametrically, Ad-shLSD1 showed an opposite effect, and more importantly, it significantly down-regulated the α-SMA levels. Furthermore, the protein levels of α-SMA, Fibronectin, collagen I and collagen IV expression were measured to evaluate the fibrogenesis degree. As shown in Fig. 4B, TGF-β1 markedly promoted LSD1, α-SMA, Fibronectin, collagen I and collagen IV expression compared with Control group, indicating the successful establishment of NRK-49F fibroblast activation. However, the effect of TGF-β1-induced cell activation was decreased by knockdown of LSD1. These findings suggested that knockdown of LSD1 suppressed NRK-49F cells activation.

### LSD1 activates TGF-β1/Smad3 pathway through suppressing SIRT3 expression

**Fig. 5:**
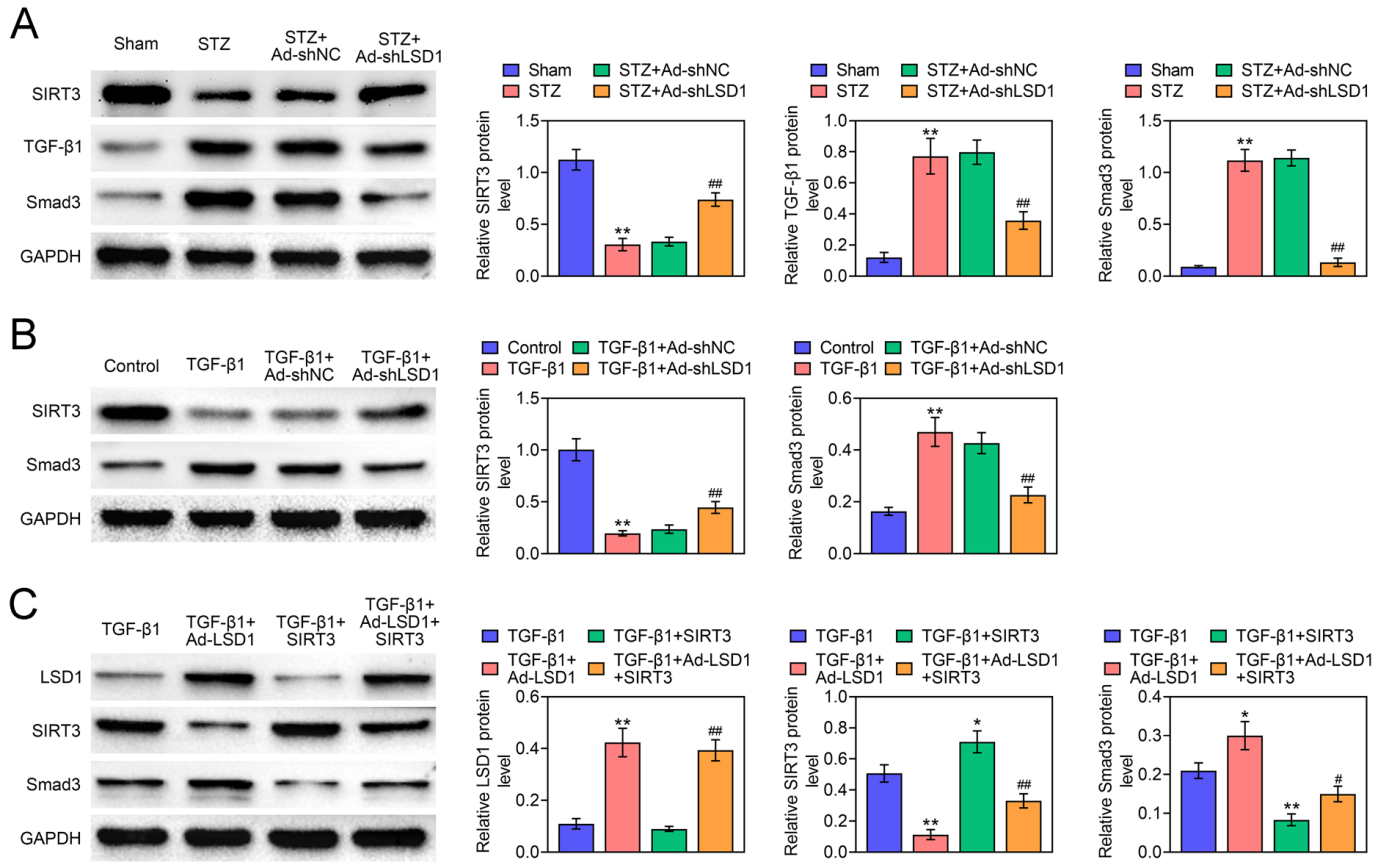
LSD1 activates TGF-β1/Smad3 pathway through suppressing SIRT3 expression. **A** The expression of SIRT3, TGF-β1 and Smad3 were determined by western blot in different rats groups. **B** The expression of SIRT3 and Smad3 were detected by western blot in different treated NRK-49F cells. The NRK-49F cells were transfected with Ad-LSD1 or SIRT3, or co-transfected with both Ad-LSD1 and SIRT3. **C** After cells pre-treated with TGF-β1, the LSD1, SIRT3 and Smad3 expression were evaluated by western blot. Each experiment repeated three times. *p < 0.05, **p < 0.01 vs. TGF-β1. ^#^*p* < 0.05, ^##^*p* < 0.01 vs. TGF-β1 + Ad-LSD1

To further confirm the mechanism by which LSD1 regulates TGF-β1/Smad3 pathway, the western blot assay was performed. In STZ-induced DN rats, STZ injection promoted TGF-β1 and Smad3 expression but reduced SIRT3 expression, whereas these changes were reversed by knockdown of LSD1, indicating that knockdown of LSD1 deactivated TGF-β1/Smad3 pathway in vivo (Fig. 5A). More importantly, STZ treatment promoted the pSMAD3 expression, which was inhibited by sh-LSD1, indicating that sh-LSD1 deactivated SMAD3 signal pathway in vivo (Additional file 1: Fig. S1A). Meanwhile, TGF-β1 suppressed SIRT3 expression but increased Smad3 expression in NRK-49F cells. Interestingly, the increased Smad3 and decreased SIRT3 levels were reversed by knockdown of LSD1, suggesting that knockdown of LSD1 deactivated TGF-β1/Smad3 pathway in vitro (Fig. 5B). Moreover, overexpression of LSD1 enhanced Smad3 expression but reduced SIRT3 expression. At the same time, co-treatment with TGF-β1 could further promote the Smad3 expression and suppress SIRT3 expression. Fortunately, the effect of overexpression of LSD1 on Smad3 could be partially counteracted by overexpression of SIRT3 (Fig. 5C). Besides, compared with TGF-ß treatment cells, overexpression of LSD1 promoted pSMAD3 expression, which was further reversed by overexpression of SIRT3, indicating that sh-LSD1 deactivated SMAD3 signal pathway in vitro (Additional file 1: Fig. S1B). In conclusion, we demonstrated that LSD1 activated TGF-β1/Smad3 pathway through suppressing SIRT3 expression.

### LSD1 induces renal fibrosis via decreasing SIRT3 expression and activating TGF-β1/Smad3 pathway

**Fig. 6:**
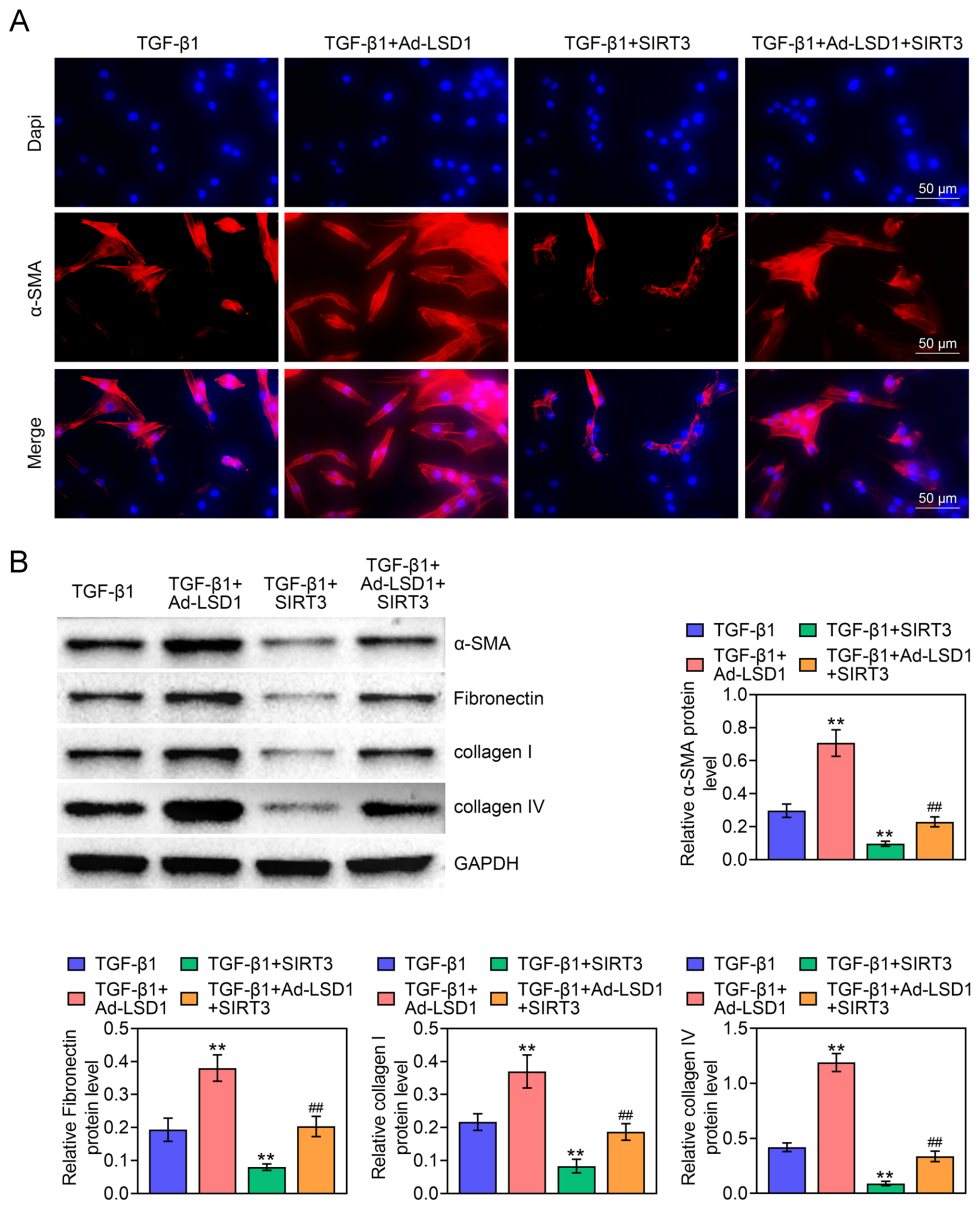
LSD1 induces renal fibrosis via decreasing SIRT3 expression and activating TGF-β1/Smad3 pathway. **A** The expression of α-SMA expression was assessed by IF assay (bar = 50 μm). **B** The expression of renal fibrosis biomarkers containing α-SMA, Fibronectin, collagen I and collagen IV were determined by western blot in NRK-49F cells. Each experiment repeated three times. **p < 0.01 vs. TGF-β1. ^##^*p* < 0.01 vs. TGF-β1 + Ad-LSD1

To investigate whether TGF-β1/Smad3 pathway was the mechanism, by which LSD1 exerted its function on NRK-49F activation, the expression levels of renal fibrosis biomarkers were evaluated. As illustrated in Fig. 6A, overexpression of LSD1 induced α-SMA expression but overexpression of SIRT3 reduced α-SMA expression, whereas the impact of overexpression of LSD1 on α-SMA expression could be partially counteracted by overexpression of SIRT3. More importantly, overexpression of LSD1 promoted α-SMA, Fibronectin, collagen I and collagen IV expression, while over expression of SIRT3 had the opposite effects. Interestingly, the function of over expression of LSD1 on renal fibrosis biomarkers was partially counteracted by overexpression of SIRT3 (Fig. 6B). Therefore, these findings indicated that LSD1 induced renal fibrosis via decreasing SIRT3 expression and activating TGF-β1/Smad3 pathway.

## Discussion

In this study, the STZ-induced DN rat model was successfully established. Knockdown of LSD1 alleviated STZ-induced renal injury. In addition, knockdown of LSD1 decreased the expression of serum biochemical markers, containing urine output (24 h), urinary protein (24 h), serum creatinine, BUN and UACR. Furthermore, we proved that knockdown of LSD1 alleviated renal fibrosis in STZ-induced DN rats. In vitro, knockdown of LSD1 suppressed NRK-49F cells activation and overexpression of LSD1 induced renal fibrosis. Moreover, knockdown of LSD1 could deactivate TGF-β1/Smad3 pathway and promote SIRT3 expression in vivo and in vitro. The rescue experiments confirmed that LSD1 induced renal fibrosis via decreasing SIRT3 expression and activating TGF-β1/Smad3 pathway. This might be the first time we confirmed that LSD1 was involved in a rat renal fibrosis, providing a reasonable strategy for developing novel drugs targeting LDS1 to block renal fibrosis.

Accumulating evidences have proved that LSD1 plays vital roles in regulating fundamental cellular processes via mediating many signaling pathways [27, 28]. LSD1 is abnormally overexpressed in acute myeloid leukemia and small lung cancer cells, and inactivation of LSD1 suppresses cancer cell differentiation, proliferation, invasion and migration [29]. In diabetes, LSD1 inhibition enhanced insulin secretion in response to glucose stimulation in insulin-producing cells [30]. LSD1 expression is up-regulated in high glucose treated cells and diabetes rats [31]. Consistent with this result, we have proved that LSD1 expression was enhanced in STZ-induced DN rats. Linda et al. have confirmed that a hypo-methylation of H3K4 at SOD2 promoter by LSD-1 increased ROS that causes diabetic retinopathy [32]. However, the role of LSD1 in DN remains unclear. DN is characterized by structural and functional abnormalities, including urinary albumin excretion, mesangial hypertrophy, and fibrosis mesangial cells in glomeruli [33, 34]. Interestingly, we demonstrated that knockdown of LSD1 alleviated STZ-induced renal injury and reduced urinary albumin excretion, which was a strongly evidence that LSD1 was involved in the occurrence of DN. Nevertheless, the mechanism of LSD1 in regulating STZ-induced DN is still unknown.

Accumulation of abnormal expression of collagen and fibronectin, and the fibroblast activation can stimulate epithelial to mesenchymal transition (EMT) and excessive accumulation of extracellular matrix components, ultimately leading to renal fibrosis [35]. Among these, TGF-β serves as the main regulator that induces EMT. In addition, TGF-β may be the most effective and pervasive profibrotic factor, acting through a variety of intracellular signaling pathways including protein kinases and transcription factors [36]. More importantly, the regulation of TGF-β in EMT rely on Smad3-dependent transcriptional regulation. Mice knockdown of Smad3 are resistant to induce EMT and show a block in EMT and a reduction in inducing TGF-β in renal tubular epithelial cells [37]. Similarly, upregulation of Smad3 synergistically enhanced the EMT response [38]. Fortunately, we have proved that LSD1 could activate TGF-β1/Smad3 pathway, providing an explanation why LSD1 induced renal fibrosis. Besides, it has been reported that LSD1 is recruited by NR4A1 to suppresse TGF-ß signaling in skin, lung, liver, and kidney fibrosis in mice, which seems to be widely divergent with our results [16]. We suspected that LSD1 activated TGF-β signal pathway and directly induced renal fibrosis, which could be recruited by NR4A1 and formed a repressor complex to limit pro-fibrotic TGF-β effects. In renal fibrosis, the direct effect of LSD1 on TGF was greater than that of NR4A1 recruitment, resulting in LSD1 inducing renal fibrosis.

TGF-β1 treatment leads to depletion of endogenous SIRT3, which is similar to our results [39]. SIRT3 is considered to be a major mitochondrial deacetylase that can help repair DN through protecting mitochondrial homeostasis by modulation of mitophagy [40]. In addition, SIRT3 can block the characteristics of organ fibrosis by regulating TGF-β/Smad signaling [22, 41]. In vivo study showed that SIRT3 suppression is associated with renal fibrosis, and further knockdown of SIRT3 profound renal fibrogenic phenotype in mice [20]. Consistent with these findings, we found that LSD1 induced renal fibrosis via decreasing SIRT3 expression and activating TGF-β1/Smad3 pathway, which is a potential mechanism of LSD1 in renal fibrosis. However, there is still various of questions need to be illustrated. LSD1 may induce renal fibrosis not only through SIRT3 but also other genes. Besides, how LSD1 regulates SIRT3 expression is still unknown. Moreover, given that SIRT3 deficiency is associated with the regulation of renal oxidative metabolism and SIRT3 suppresses mitochondrial biosynthesis and metabolism, whether LSD1 can regulate renal oxidative metabolism and mitochondrial biosynthesis and metabolism and then participates in the occurrence of DN remains unknown [42, 43]. These hypotheses need to be investigated in the future.

## Conclusion

Our finding indicated that LSD1 deficiency alleviates STZ-induced renal injury. Overexpression of LSD1 induces renal fibrosis via decreasing SIRT3 expression and activating TGF-β1/Smad3 pathway. This study provides a new therapeutic approach in DN treatments.

## Supplementary Information

**Additional file 1.** sh-LSD1 deactivated SMAD3 signal pathway *in vivo* and *in vitro*.

## Data Availability

All data generated or analyzed during this study are included in this published article.
