# Supplementary figures and images for: Histone lysine-specific demethylase 1 induced renal fibrosis via decreasing sirtuin 3 expression and activating TGF-β1/Smad3 pathway in diabetic nephropathy
Source: Diabetol Metab Syndr. 2022 Jan 4;14:2. doi: 10.1186/s13098-021-00771-z (PMC8725532; doi:10.1186/s13098-021-00771-z)

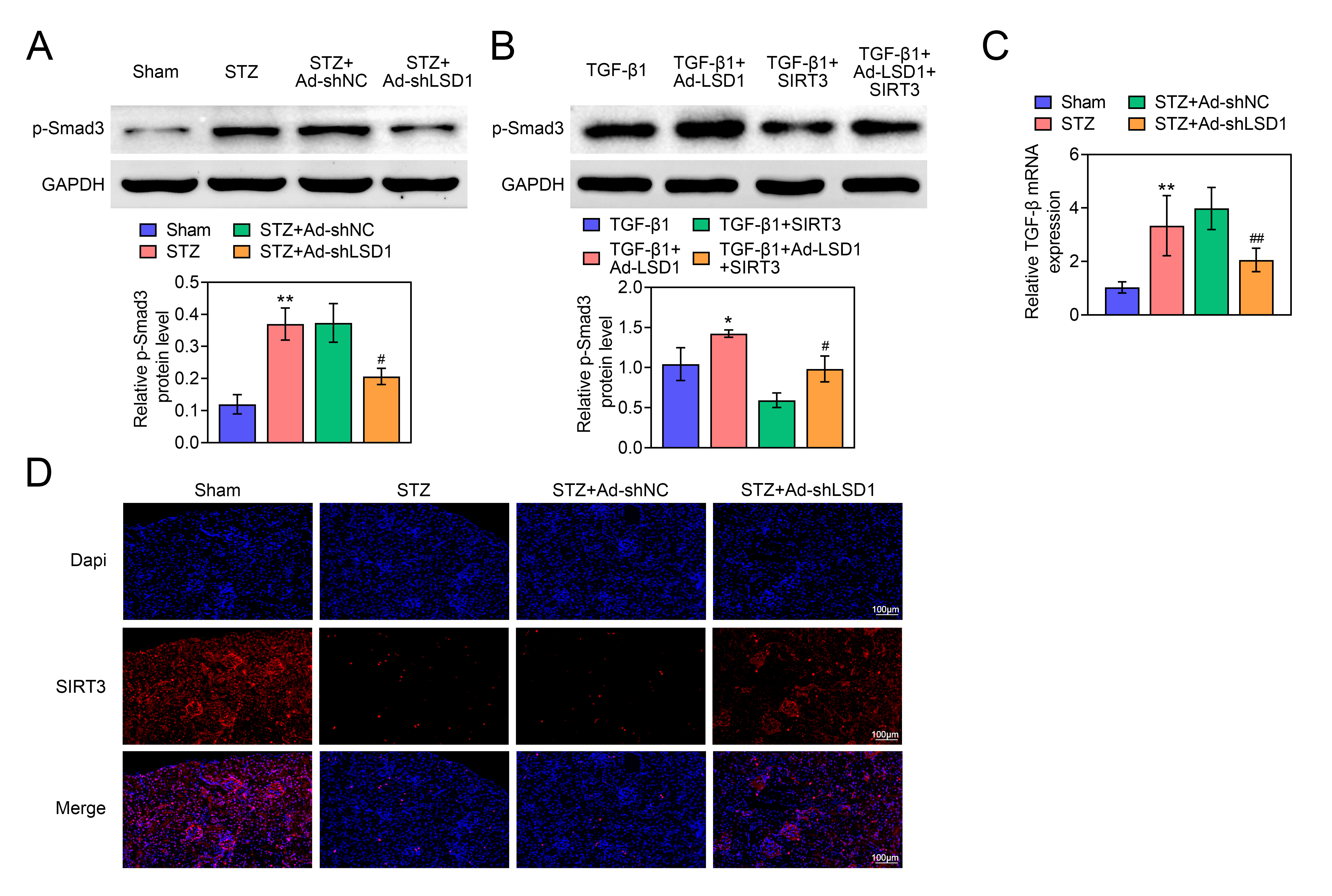

Supplement: Supplementary file 1 — Additional file 1. sh-LSD1 deactivated SMAD3 signal pathway in vivo and in vitro. [file 13098_2021_771_MOESM1_ESM.jpg]
